# A technical breakthrough close at hand: feasible approaches toward establishing a gene-targeting genetic transformation system in seaweeds

**DOI:** 10.3389/fpls.2014.00498

**Published:** 2014-09-24

**Authors:** Koji Mikami

**Affiliations:** Laboratory of Aquaculture, Genetics and Genomics, Division of Marine Life Science, Faculty of Fisheries Sciences, Hokkaido UniversityHakodate, Japan

**Keywords:** gene expression, gene targeting, genome, genetic transformation, seaweed

The lack of a genetic transformation system that allows integration of a foreign gene into the genome is a serious hindrance to progress in biological research on seaweeds. In contrast, physiological research in seaweeds has a long history of focus on the establishment of cell polarity and multicellularity (Brownlee and Bouget, 1998; Hable and Hart, 2010). Today, nuclear genomes have been recently sequenced for many species of seaweeds and there is now a significant accumulation of genomic information in the form of expressed sequence tag data in public and private databases for analyses (see Mikami, 2014). Although physiological and molecular biological approaches have the potential to allow genome-wide and comprehensive analyses of factors involved in seaweed development, the lack of a genetic transformation system hinders functional identification of genes encoding regulatory components of seaweed development. This is impeding the application of the molecular biological knowledge in industrial and economic activities related to seaweed. Because genetic manipulation systems are indispensable for the functional analysis of genes, it is necessary to develop reverse genetic methods for gene knock-out, knock-in, or replacement in a variety of seaweeds via either homologous recombination or random integration of foreign DNA with a marker to identify its integration site in the genome.

To accelerate progress toward establishing genetic transformation methods, there are four important issues to be resolved: (1) creating expression constructs particularly suited to seaweed cells; (2) transferring the expression constructs into cells; (3) ensuring integration of expression constructs into the genome; and (4) selecting genetically transformed cells. It has already been confirmed that combining codon-optimized coding regions and endogenous strong promoters results in high-efficiency expression in seaweed cells (Mikami et al., 2011; Mikami and Uji, 2012; Mikami, 2013). Seaweed genomes generally have high GC content and thus coding sequences should be biased toward higher amounts of G and C. Moreover, the *Cauliflower mosaic virus* (CaMV) 35S RNA promoter that has been used for foreign gene expression in seaweeds prior to establishment of the transient gene expression has rather low activity in seaweed cells (Mikami et al., 2011; Mikami and Uji, 2012; Mikami, 2013). But importantly, neither the codon optimization nor endogenous promoters yet tested could enhance the expression of foreign genes in seaweeds (Mikami et al., 2011). These findings clearly indicate that codon-optimized coding regions directed by strong endogenous promoters will be required elements of the design of constructs that can be expressed efficiently in seaweed cells (Mikami et al., 2011; Mikami and Uji, 2012; Mikami, 2013). This strategy has already been applied to a wide variety of red seaweeds (Hirata et al., 2011a,b; Son et al., 2011). Moreover, the second requirement for genetic transformation, to transfer expression constructs into cells, has been resolved using particle bombardment technology in red seaweeds (Mikami et al., 2011; Mikami and Uji, 2012; Mikami, 2013) and microinjection in *Ectocarpus siliculosus* (Farnham et al., 2013). However, neither electroporation nor glass bead methods have yet been successful for transferring DNA constructs into seaweed cells. Our preliminary data indicated that the glass bead method and various gene transfer regents designed for mammalian cells were ineffective for cell wall–less protoplasts and monospores from the red seaweed *Pyropia yezoensis*.

Taken together, the third and fourth issues—to ensure integration of expression constructs into the genome, and select genetically transformed cells, respectively—still have to be resolved to establish nuclear genetic transformation.

## Insertion of a foreign gene into the genome with an integration site marker

Gene targeting is a fundamental reverse-genetic approach to disrupt genetic information to allow direct examination of the function of genes of interest. So far, nuclear genetic transformation via high frequency homologous recombination has been established only for the red microalga *Cyanidioschyzon merolae* (Minoda et al., 2004) and the oil-producing heterokont eustigmatophyte *Nannochloropsis* (Kilian et al., 2011). However, in *Chlamydomonas reinhardtii*, a stable type of non-integrated transformation based on plasmid transfection is usually employed because of quite low frequency of homologous recombination (Zorin et al., 2005). Thus, it is important to elucidate whether seaweeds are competent to perform homologous recombination. To this end, as illustrated in a figure by Weeks (2011), approximately 2–3 kb of genomic DNA containing the exons and introns corresponding to a target gene is amplified and then divided into two parts, both 1–1.5 kb long, by digestion with an appropriate restriction enzyme. Then the chosen promoter-coding region is flanked on each side by these fragments. Before transferring such a gene-disruption construct into cells by particle bombardment or microinjection, linearization of the plasmid DNA construct is sometimes required. Indeed, the transformation efficiency of *Nannochloropsis* sp. with a circular plasmid was quite low (Kilian et al., 2011). This type of approach could confirm whether seaweeds have the ability to perform homologous recombination and also whether the gene disruption has occurred at the correct site in the genome. Both genomic PCR to amplifying the integration sites and Southern blot analysis to check the copy number of the integrated fragment are required for checking the successful homologous recombination.

When homologous recombination is not possible, *Agrobacterium*-mediated transformation can be a suitable next choice. Although *Agrobacterium*-mediated gene integration is random, insertion is expected to occur in an exon or an intron of a gene, resulting in the creation of gene-specific knockout mutants that inactivate particular genes involved in physiological regulation. Cheney et al. (2001) reported *Agrobacterium*-mediated genetic transformation in the red alga *P. yezoensis* at a conference; however, no subsequent report of the success of this approach has yet appeared for any other seaweed. Because Cheney et al. (2001) used the CaMV 35S RNA promoter and bacterial AT-rich β-glucronidase (GUS) coding sequence, weak expression of the GUS gene might not have been detectable. Thus, it is worthwhile to consider attempting *Agrobacterium*-mediated genetic transformation using the combination of a strong endogenous promoter and the codon-optimized coding region of a reporter. Once this method has become established, a series of T-DNA insertion mutants can be made, some of which would indicate gene targeting and visible phenotypes related to developmentally regulated processes.

## Selection of genetically transformed cells

Isolation of genetically transformed cells is quite important for the production of transgenic lines. For the red unicellular alga *C. merolae*, 5-fluoroorotic acid (5-FOA)-resistant and uracil-auxotrophic mutants were used as recipients for genetic transformation (Minoda et al., 2004; Ohnuma et al., 2008). The system is based on the production of toxic 5-fluorouracil by orotate phosphoribosyltransferase (URA5) and orotidine-5′-phosphate decarboxylase (URA3), both of which are encoded by a single gene as one open reading frame of uridine monophosphate synthetase (UMPS) in *C. merolae* (Minoda et al., 2004). Minoda et al. (2004) indicated that *C. merolae* cells are sensitive to 800 μg 5-FOA, thus cells strains that are both 5-FOA-resistant and uracil auxotrophic could be isolated. Because the UMPS gene with the same structure as that from *C. merolae* is highly conserved in red, green, and brown algae (unpublished), it is possible that if 5-FOA-resistant uracil auxotrophic strains are obtained in a variety of other seaweeds, a genetic transformation system such as that in *C. merolae* could be established for each. However, preliminary data clearly indicate that there is substantial intrinsic 5-FOA-resistance in *Bangia fuscopurpurea* and it would therefore be impossible to develop a UMPS-dependent transformation system, at least in this seaweed. Thus, it will be necessary to assess the degree of 5-FOA-sensitivity of other seaweeds.

Selection methods based on herbicide-resistant phenotypes have already established in higher plants (Shimizu et al., 2011). Acetohydroxyacid synthase (AHAS; also known as acetolactate synthase), which catalyzes the first step in biosynthesis of branched-chain amino acids such as valine, leucine, and isoleucine (Duggleby et al., 2008), is a target of at least three classes of herbicides, including pyrimidinyl carboxylate (PC), sulfonylurea (SU), and imidazolinone herbicides (Kawai et al., 2008; Shimizu et al., 2008). Among these, sulfometuron methyl (SMM) and bensulfuron methyl (BM) (both SU), and bispyribac-sodium, pyrithiobac-sodium, and pyriminobac-methyl (all PC) have been used to screen for genetically transformed cells, for which inhibitor-tolerant variants of AHAS were used as selectable markers (Lapidot et al., 2002; Shimizu et al., 2008; Kawai et al., 2010). Thus, if seaweeds are sensitive to AHAS inhibitors, selection of transformed cells could then be possible, as in the report of successful chloroplast transformation in the red unicellular alga *Porphyridium* sp. using SMM sensitivity (Lapidot et al., 2002). However, preliminary experiments indicate that *P. yezoensis* is not sensitive to SMM and bispyribac-sodium, which suggests that it might not be possible to develop herbicide-resistance systems for selection of transformants in seaweeds.

The findings above indicate that using antibiotics will be pragmatic. Doses of 2.0 mg/ml hygromycin, chloramphenicol, or paromomycin, or of 1.0 mg/ml G418, were lethal to *P. yezoensis* gametophytes, although these gametophytes were also highly resistant to ampicillin and kanamycin (Takahashi et al., 2011). Thus, as mentioned in Mikami (2013), these four antibiotics and their corresponding resistance genes are thought to be suitable for the selection of genetically transformed cells from *P. yezoensis* gametophytes. In this case, optimization of codon usage and the use of strong endogenous promoters will likely be necessary for the functional expression of the antibiotic resistance genes, according to knowledge gained from development of the transient transformation system (Mikami et al., 2011; Mikami and Uji, 2012; Mikami, 2013). At present, antibiotic-dependent selection is the most plausible method for selection of transformed seaweed cells; thus, it will be important to test the antibiotic-sensitivity of seaweeds of interest.

## Genome editing: the next generation system for gene targeting modification

Another candidate technology for a gene-targeting seaweed transformation method is genome editing. This novel gene knockout system is based on targeted double-strand breaks induced by artificial nucleases such as the zinc finger nuclease (ZFN), transcription activator-like effector nuclease, and the clustered regulatory interspaced short palindromic repeat/CRISPR-associated nuclease 9 (CRISPR/Cas9), and subsequent repair of the breaks via homologous recombination or non-homologous end joining (NHEJ) in the genome (Gaj et al., 2013; Lozano-Juste and Cutler, 2014). During NHEJ, sequence modifications occur due to the change and insertion of nucleotides, which result in gene knock-out mutants. Because this method is thought to be applicable to many species for which homologous recombination is not known, genome editing seems to be a promising method for genetic transformation of seaweeds.

At present, the ZFN genome editing method has been successfully used for targeted mutagenesis in algae only in *Chlamydomonas reinhardtii* (Sizova et al., 2013), while there has yet been no report of the application of genome editing in seaweeds. Among these three methods, the CRISPR/Cas9 system is simple and applicable to a variety of eukaryotes like yeast, animals, and plants (DiCarlo et al., 2013; refs in Gaj et al., 2013; Lozano-Juste and Cutler, 2014). To perform genome editing using CRISPR/Cas9 in seaweeds, it will be necessary to isolate a DNA fragment containing the promoter region of the U6 small RNA gene, a class III gene transcribed by RNA polymerase III, to express a fusion of the target sequence together with a guide RNA (gRNA). In addition, optimization of the codon-usage of the Cas9 endonuclease gene coding region and use of a strong promoter from the seaweed genome would allow more efficient expression of the modified Cas9. The U6 small RNA promoter-target sequence-gRNA and the endogenous promoter-modified Cas9 coding region are typically introduced into the nucleus via *Agrobacterium*-mediated transformation in plants. Thus, genome sequences of seaweeds are being carefully surveyed to identify the U6 small RNA genes and also strong promoters, but the prospects for using *Agrobacterium* for transformation of seaweeds would need to be tested.

## Conclusions

A number of paths that could be taken to establish a gene-targeting system for seaweeds have been described above. The two most critical technological issues to address include the stable integration of targeted foreign genes into the genome, and effective antibiotic-based selection of genetically transformed cells. Gene targeting may also be possible via genome editing with gene-specific artificial endonucleases; however, much would be necessary to optimize this type of system for seaweeds. Currently, the most feasible way to establish a system for genetic transformation is to insert foreign genes into the genome by site-specific homologous recombination after introduction of constructs using particle bombardment or microinjection methods and subsequent antibiotic-dependent selection of genetically transformed cells.

### Conflict of interest statement

The author declares that the research was conducted in the absence of any commercial or financial relationships that could be construed as a potential conflict of interest.
